# Use of Stacked Layers of Electrospun L-Lactide/Glycolide Co-Polymer Fibers for Rapid Construction of Skin Sheets

**DOI:** 10.3390/bioengineering8010007

**Published:** 2021-01-07

**Authors:** Mervyn Merrilees, Neil Buunk, Ning Zuo, Nigel Larsen, Samaneh Karimi, Nick Tucker

**Affiliations:** 1 Department of Anatomy and Medical Imaging, University of Auckland, Auckland 1023, New Zealand; n.zuo@auckland.ac.nz; 2 Electrospinz Limited, 44 Lee Street, Blenheim 7201, New Zealand; neil@electrospinz.co.nz; 3 Canterbury Agriculture & Science Centre, The NZ Institute for Plant & Food Research Ltd. Lincoln, Gerald Street, Lincoln 7608, New Zealand; nigellarsen1@gmail.com (N.L.); info@arianabraving.com (S.K.); NTucker@lincoln.ac.uk (N.T.)

**Keywords:** electrospun mesh, layered scaffolds, culture, fibroblasts, keratinocytes, skin

## Abstract

This paper describes a novel method for the rapid construction of skin, using multiple layers of aligned electrospun fibers as starting scaffolds. Scaffolds were spun from biodegradable L-lactide/glycolide (molar ratio 10:90) with predominantly parallel arrays of fibers attached peripherally to thin 304 stainless steel layer frames. Each layer frame was held between two thicker support frames. Human skin cells were seeded onto multiple (three–nine) scaffolds. Dermal fibroblasts were seeded on both sides of each scaffold except for one on which keratinocytes were seeded on one side only. Following 48 h of culture, the scaffolds and layer frames were unmounted from their support frames, stacked, with keratinocytes uppermost, and securely held in place by upper and lower support frames to instantly form a multilayered “dermis” and a nascent epidermis. The stack was cultured for a further 5 days during which time the cells proliferated and then adhered to form, in association with the spun fibers, a mechanically coherent tissue. Fibroblasts preferentially elongated in the dominant fiber direction and a two-dimensional weave of alternating fiber and cell alignments could be constructed by selected placement of the layer frames during stacking. Histology of the 7-day tissue stacks showed the organized layers of fibroblasts and keratinocytes immuno-positive for keratin. Electron microscopy showed attachment of fibroblasts to the lactide/glycolide fibers and small-diameter collagen fibers in the extracellular space. This novel approach could be used to engineer a range of tissues for grafting where rapid construction of tissues with aligned or woven layers would be beneficial.

## 1. Introduction

The aim of this study was to develop a new strategy for the rapid culture of bilayered skin (epidermis plus dermis) on a supportive but minimal and biodegradable scaffold for potential use as a graft. Numerous approaches are currently available for restoring cover to skin wounds including the use of autologous cultured keratinocytes and dermal fibroblasts and the use of these cells on acellular scaffolds [1,2,3,4]. Some approaches have used a layered approach with separate scaffolds for the epidermis and dermis, and in some studies, for the hypodermis, followed by assembly to form a cultured skin [4]. Production of skin sheets, however, generally takes several weeks [5,6], especially if the aim is to allow time for the dermal fibroblasts to produce an extracellular matrix that includes collagen fibrils with sufficient strength for tissue integrity and surgical manipulation. Culture of cells on substantial scaffolds can shorten the time for graft production by providing early mechanical integrity, but these scaffolds may isolate fibroblasts from the mechanical forces that provide signals for the formation of an appropriate and organized extracellular matrix. Biodegradable scaffolds, however, can allow for the transfer of mechanical forces from the scaffold to the newly produced extracellular components, although if inflammation, even at low levels, accompanies the degradation, the ensuing proteoglycan-rich matrix inhibits the formation of larger-diameter and aligned collagen fibrils best able to transmit tensional forces [7,8]. The formation of elastic fibers is also inhibited by proteoglycans such as versican [7,9]. Of further importance is the organization of collagen bundles and elastic fibers. The dermis has an anisotropic weave of collagen bundles and elastic fibers that align with the lines of skin tension, or Langer’s lines. The importance of tensional forces is demonstrated by the healing of skin wounds. Incisions across the lines of tension result in formation of hypertrophic scars, whereas incisions parallel to lines of tension result in minimal scarring [10].

Numerous studies have demonstrated that skin cells can be grown on a variety of scaffolds including electrospun fibers [4,11]. The advantage of spun fibers as a starting substrate is that both the alignment and the density of fibers can be controlled. Dense scaffolds of closely packed and randomly oriented fibers, however, while providing underlying support for potential grafts, have the disadvantage of not providing early orientation signals for cells, particularly the dermal fibroblasts. Orientation is important for the production and deposition of a collagenous fibrous extracellular matrix [12]. Less dense and thin scaffolds of small-diameter spun fibers, however, which have the potential to direct the orientations of attached cells, do not provide sufficient mechanical strength for grafting until such time as the cells proliferate, multilayer, and produce an extracellular matrix. In an attempt to overcome these limitations, we have investigated a new approach to the culture of skin on electrospun fibers.

In this study, our aims were several-fold: to minimize the amount of scaffold material required to support cell attachment, growth, and elongation, to devise a layering system to shorten the culture time to achieve a multilayered dermis, to design a dermis with differing cell orientations in different layers, and to provide for early transfer of mechanical loads from the scaffold to the cells and matrix by using a very low density of small-diameter biodegradable fibers.

We report that skin can be constructed over a period of one week by seeding multiple layers of minimal scaffolds of poly(L-lactide-co-glycolide) (PLG) electrospun fibers with dermal fibroblasts and keratinocytes, then stacking the layers to instantly create a skin sheet. This novel approach allowed for the construction of skin sheets with sufficient integrity to be manipulated with surgical instruments. This stacking of layers during the early culture also allowed for different alignments of dermal fibroblasts at different depths, mimicking more closely the fibroblast alignments seen in normal dermis.

## 2. Materials and Methods

### 2.1. Electrospinning

The aligned fiber arrays used in the work described in this paper were made using a proprietary technology, developed from a proposal by Nurfaizey et al. [13]. The method addresses the twin difficulties of control of production rate and fiber handling, using a hybrid approach combining passive gap spinning with controlled variation in the electrostatic field. The production of oriented fibers allows the control of the degree of isotropy of the mechanical properties of the scaffolds to match those of the target tissues (this facet of the work will be fully described elsewhere). In addition, the manufacturing cycle is automated, being run using an Omron programmable logic controller: the current pilot-scale plant is readily scalable for industrial production. To cope with the high rate of solvent diffusion, the silicone pipework of the Electrospinz Dorris electrospinner was replaced with 316 grade stainless steel pipes. The apparatus allows the deposition of a controlled number of highly aligned fibers. To facilitate handling, the fibers were deposited directly on to 25 µm layer frames (304 stainless steel) and then press-welded in position. The frame-mounted fibers were then sterilized using low-temperature hydrogen peroxide gas plasma (the Sterrad^™^ process) before being packed for shipment in a 280 mm × 400 mm metallized foil pouch (3 seal silver foil pouch, Cas-Pak Products Ltd., Silverdale, New Zealand), which was evacuated and sealed using an A300/16 Multivac vacuum sealer (Sepp Haggenmüller GmbH & Co, Wolfertschwenden, Germany). Purac Purasorb PLG 1017, a GMP grade of L-lactide/glycolide co-polymer with a component molar ratio of 10:90, was used to manufacture the spun fibers. This material is used in the manufacture of biodegradable sutures and was chosen after direct consultation with Messrs. Purac Asia Pacific PTE Ltd (Singapore). based on considerations of commercial availability, speed of bio-absorption, certification for human implantation, and the recommendation of the manufacturer based on feedback they had received for the general suitability of the material for electrospinning. The spinning dope was prepared as an 8 wt% solution in Hexafluoroisopropanol (HFIP).

### 2.2. Cell Culture

Human neonatal Dermal Fibroblasts (hnDF) and human neonatal primary Keratinocytes (hnK) from ATCC^®^ Primary Cell Solutions™ were cultured according to protocols provided (refer to ATCC^®^ PCS-201-010 and ATCC^®^ PCS-200-010). Fibroblasts were cultured in DMEM-high glucose medium (Invitrogen, Carlsbad, CA, USA, No. 10569-044), supplemented with 10% FBS (Thermo HyClone, Logan, UT, USA, No. SH30406.02) and glutamine pen-strep (Invitrogen, Carlsbad, CA, USA, No.10378-016). Keratinocytes were cultured in EpiLife Medium (Invitrogen, Carlsbad, CA, USA, No. M-EPI-500-CA) with Human keratinocyte Growth Supplement (HKGS, Invitrogen, Carls-bad, CA, USA, No.S-001-5). In cultures in which both cell types were present (i.e., on stacked layer frames), cells were cultured in DMEM-high glucose medium (Invitrogen, Carlsbad, CA, USA, No. 10569-044) supplemented with HKGS (1%).

**Figure 1:**
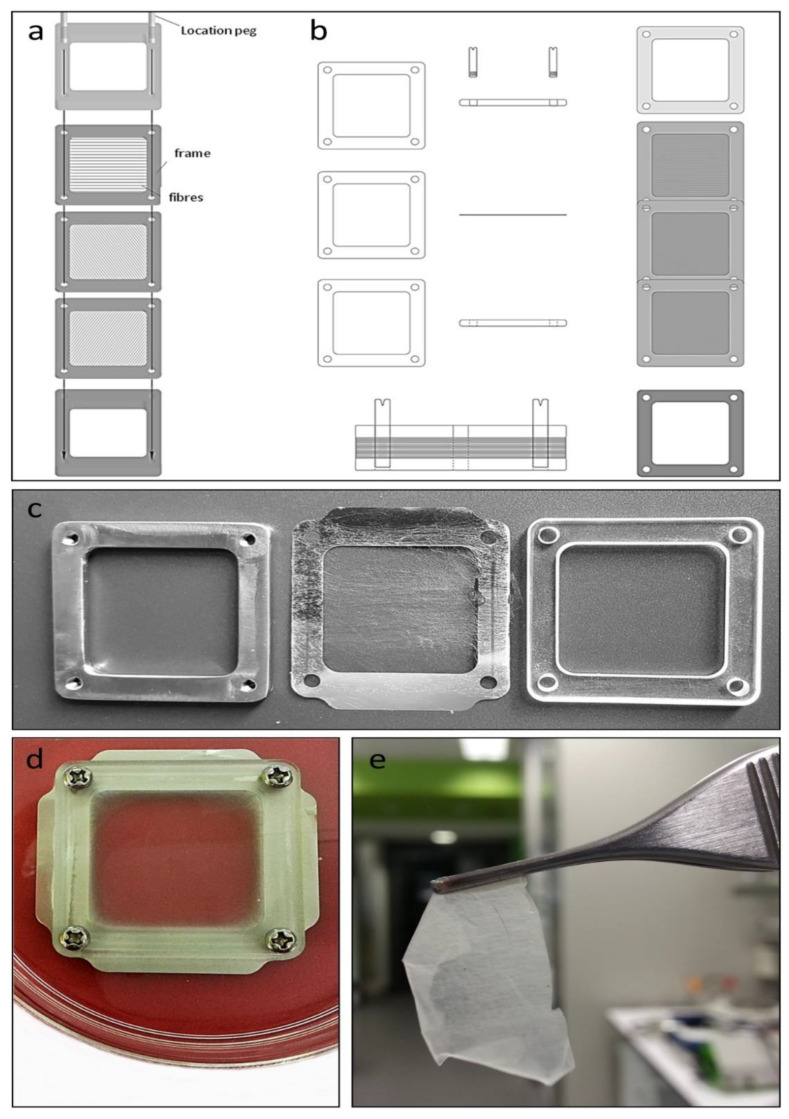
Layer frame construction. (**a**,**b**) Diagrammatic representation of support and layer frames with electrospun fibers. (**a**) Upper support frame is constructed of clear 3 mm thick Poly(methyl methacrylate) plastic, the middle 3 layer frames of 25 µm 304 grade stainless steel, and the lower support frame of 3 mm thick 304 stainless steel. Support frames are 4.4 cm × 4.4 cm overall. Fibers are deposited across stainless steel layer frames and used individually as substrates for cultured cells. Cells are seeded on the upper side of each layer frame and 24 h later, layer frames are turned over and seeded again. After a further 24 h, the seeded layer frames are stacked and sandwiched between the plastic support frame and the stainless steel frame using the location pegs (stainless steel) for alignment as shown in (**b**). (**c**) Left: lower support frame (304 stainless steel); middle: single stainless steel layer frame with electrospun PLG mesh; right: upper support frame (plastic). (**d**) Stacked layer frames (9 in total), seeded with cells and cultured for 7 days, immersed in culture medium in 100 mm culture dish. The stacked layer frames were constructed with two (**c**) or four (**d**) extension flanges, visible outside the margins of the support frames, to allow for handling with forceps. (**e**) Multilayered fiber and cellular skin sheet cut from stack of support and layer frames in (**d**), following 7 days in culture.

**Figure 2:**
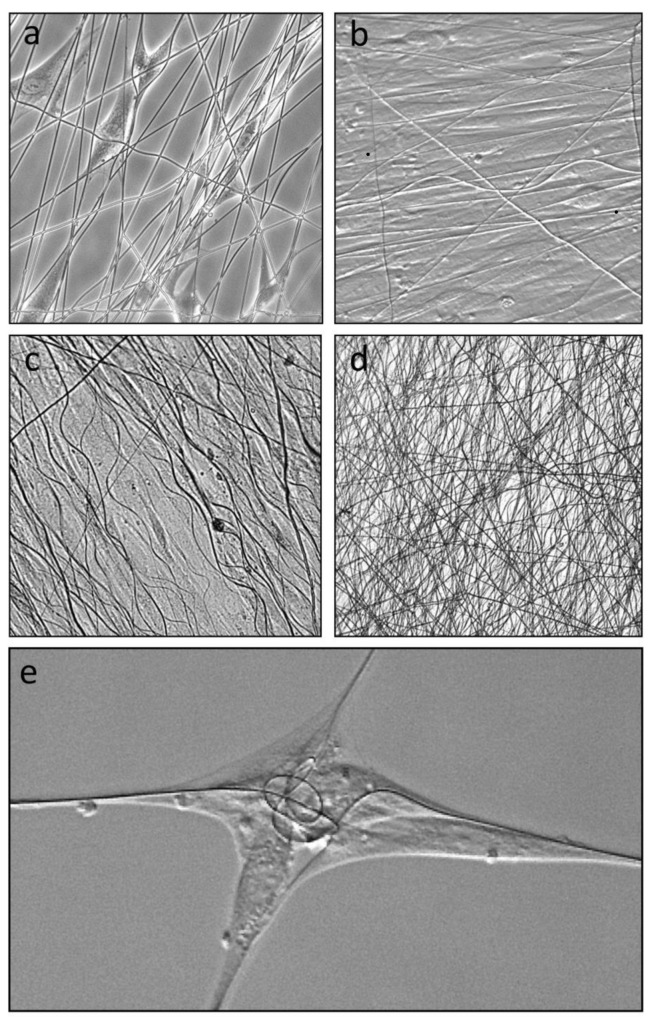
(**a**) Electrospun PLG fibers (1–2 µm diameter) of a single layer frame seeded with dermal fibroblasts, on one side, and imaged with phase contrast at 24 h showing attachment and alignment of scattered cells along fibers. (**b**) Single layer frame on day 4 showing confluent sheets of fibroblasts attached to and supported by fibers. Phase image shows cells aligned (horizontally) in the same direction as the majority of fibers. (**c**) Brightfield image of fibers and cells on a single layer frame at day 7. Fibers are aligned in the same direction as cells. Wavy fiber profiles indicate removal of tension on individual fibers. (**d**) Brightfield image of three layers of stacked layer frames, cultured for 7 days, showing alternating orientations of fiber meshes in the different layers. (**e**) Cluster of four cells, on day 2, attached to two crossed fibers showing fibers drawn into coils by extended cells.

Individual layer frames (Figure 1a,b), with attached spun fibers (Figure 1c), were prepared for culture by immersing each layer frame in fibroblast medium in a 100 mm culture dish (Falcon^™^, Glendale, AZ, USA, No. FAL353003) overnight, followed by the mounting of each layer frame between two support frames, an upper frame of clear 3 mm thick Poly(methyl methacrylate) plastic, and a lower frame of 3 mm thick 304 stainless steel (Figure 1c), with all layers held together with four stainless steel screws. Media were replaced to a level whereby the electrospun fibers were immersed but with the upper surface of the upper support frame slightly above the surface of the medium, creating a contained pool of medium above the mesh into which cells were seeded. Fibroblasts (1.8 × 10^6^ cells in 1 mL) were seeded directly into the medium above the mesh and left to settle and attach overnight. The seeding density was determined empirically, with the high number of cells seeded inside the support frames (~8 sq cm) allowing for some cells to pass through the open mesh (see Figure 2a) and not attach to fibers. Multiple layer frames were seeded depending on the number of layers intended for the final skin sheet. Initially, we produced skin sheets of two and three layers of mesh, and while the cells and their organization were similar to thicker sheets with more layers, these skin sheets at 7 days were delicate and more difficult to manipulate. The number of layers yielding a skin sheet with sufficient strength to be handled with surgical instruments was found to be 6 to 9 layers.

The day following the first seeding, all except one of the mounted layer frames were turned over and seeded again with fibroblasts. The remaining mounted layer frame was then turned over and seeded with keratinocytes (1.0 × 10^6^ each frame). The following day, all layer frames were unmounted from their support frames and transferred one by one onto a new sterile lower metal support frame and stacked on top of each other using two location pegs inserted into the lower support frame to align the layer frames during stacking. The final layer frame placed in the stack contained keratinocytes on the upper surface of the mesh. Two fixing screws were then used to secure a new sterile upper plastic support frame to the lower metal support frame, compressing the multiple layer frames, followed by removal of the two location pegs and replacement with two more fixing screws. The entire unit was then submerged in media in a 100 mm dish (Figure 1d) and cultured for a further 5 days for a total culture time of 7 days. Following culture, the adherent multilayered construct was cut from the mounted layer frames with surgical scissors (Figure 1e) and processed for microscopy.

### 2.3. Microscopy

Skin sheets on day 7 were fixed in 4% paraformaldehyde for 30 min and processed either for paraffin embedding and sectioning for histological analysis and immunostaining, or for resin embedding and thin sectioning for electron microscopy. Paraffin sections were stained with hematoxylin and eosin and immunostained for keratin. For the latter, de-paraffinized sections were washed in PBS, blocked in 1% donkey serum and incubated for 2 h with primary Keratin antibody C11 (1:50; Thermo Fisher Scientific NZ Ltd, Auckland, New Zealand; SCZSC-8018), washed in PBS, and incubated for 1 h in secondary antibody Alexa 488 goat-anti-mouse IgG at 1:500. Slides were rinsed in PBS and mounted with ProLong^®^ Gold Antifade Reagent containing DAPI. Paraformaldehyde-fixed skin sheets for electron microscopy were post fixed in 1% OsO_4_, processed, and sectioned transversely through the multilayers of cells and mesh. Thin sections, stained with uranyl acetate and lead citrate, were viewed and photographed on a Tecnai^™^ G^2^ Spirit Twin transmission electron microscope.

## 3. Results

Dermal fibroblasts seeded onto submerged layer frames with attached electrospun L-lactide/glycolide co-polymer mesh quickly attached and elongated along individual fibers (Figure 2a). Where cells encountered crossed or angled fibers, they frequently simultaneously attached to two or more fibers, most clearly seen with cells attached to the very low fiber density mesh. Continued culture of single layer frames for 4 days showed that the fibers supported confluent cell sheets (Figure 2b), with cells aligned to match the predominant alignment of the underlying fibers. Notably by day 4, some fibers were no longer extended but assumed a sinusoidal wave pattern (Figure 2b), which was more developed by day 7 (Figure 2c), indicating removal of tension on the fibers between cell attachment points. Wave patterns were less evident in stacked layer frames where fiber layers could be stacked to create a trellis-like web (Figure 2d). It was noted that fibroblasts attached to sparse fibers on single layer frames were able to significantly shorten and, in some cases, coil (Figure 2e) the fibers, indicating that cells attached to fibers were able to generate tensional forces.

To create skin with a dermis and epidermis, fibroblasts were seeded on alternate days on each side of individual layer frames, and keratinocytes were seeded on one side of one layer frame on the second day, and all layer frames stacked together at the beginning of day three, with keratinocytes uppermost, as described in the Methods. Two-, three-, six-, and nine-layered skin sheets were constructed and cultured until day 7. The six- and nine-layered sheets were translucent in media (Figure 1c) and, when cut from the support frames, had sufficient integrity for manipulation with forceps (Figure 1d). By day 9 and 10 in culture, the biodegradable L-lactide/glycolide co-polymer fibers showed signs of degradation and from 10 to 13 days, some breakage occurred on the skin sheets, often at the same point across all stacked layers, suggesting propagation due to reduced mechanical strength throughout the layers.

**Figure 3:**
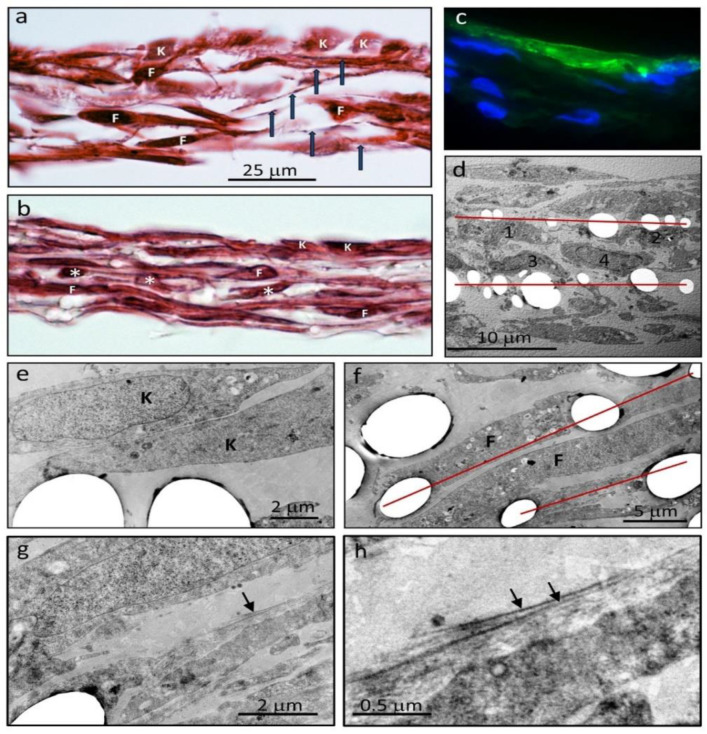
Histology and ultrastructure. (**a**) Histological section of skin layer formed from six stacked layer frames, cultured for 7 days, stained with hematoxylin and eosin. Five layer frames were seeded with dermal fibroblasts (F) on each side of the fiber mesh before stacking. The sixth layer frame was seeded with dermal fibroblasts on one side and 24 h later with keratinocytes (K) on the other side. During assembly of the layer frames, the layer frame with keratinocytes was placed on top with keratinocytes uppermost. The arrows point to the six layers of PLG fibers, stained due to cell processes extending along fibers. The separation between the six layers is due to the sampling location close to the inner margins of the stacked stainless steel layer frames. (**b**) Similar image to (**a**), but from a location at a distance from the frame margin showing a more compact arrangement of the six layers. Asterisks mark cells in a layer in which cells have been sectioned across their long axis, in contrast to the cells immediately below which are cut longitudinally. (**c**) Three-layered skin sheet immuno-fluorescently stained (green) for keratin and for cell nuclei (DAPI, blue), showing keratin deposits in upper layer. (**d**) Low-power electron micrograph of a two-layered skin sheet showing positions of dissolved PLG electrospun fibers within the skin sheet, with the plane of individual fibers of variable size marked by the red lines. Fibroblasts labeled 1 and 2 were seeded onto the underside of the upper mesh of fibers; fibroblasts labeled 3 and 4 were seeded on the top side of the lower mesh of fibers. The cells above the upper red line are keratinocytes. (**e**) Electron micrograph of keratinocytes (K) above upper layer of fibers. (**f**) Fibroblasts (F) in a multilayered skin sheet. The fibroblast marked F above the upper red line is attached to and spans two fiber elements. (**g**) Deposition of collagen fibrils (arrowed) on the edge of the pericellular matrix, and shown at higher magnification in panel (**h**).

Histological sections of the skin sheets (Figure 3a,b) showed a layer of keratinocytes, immune-positive for keratin (Figure 3c), and multilayers of fibroblasts. The L-lactide/glycolide co-polymer mesh did not stain with hematoxylin and eosin but cell extensions along the fibers were visible and marked the position of each layer of fibers. The individual layers were most clearly discernable near the edges of the stacked layer frames where the 25 micron-thick stainless steel layer frames provided for separation of the layers (Figure 3a). Towards the center of the stacked meshes, the layers of cells formed a more compact tissue, and the cell density was correspondingly increased (Figure 3b). Alternating profiles of fibroblasts cut longitudinally and in cross-section in adjacent layers of the dermis were also evident, due to the different underlying fiber directions and fibroblast elongation along aligned fibers (Figure 3b).

The arrangement of cells and relationship to the fibers were more clearly seen by electron microscopy (Figure 3d–f). The fibers were removed by the processing solvents, but their location and profiles were clearly evident. Notably, the volume of the fiber support framework was a relatively small fraction of the tissue volume (11% in Figure 3d) and the position of the cells relative to the fibers in each layer was clearly evident, with cells seeded on each side of individual layer frames retaining their relative positions (Figure 3d). Both keratinocytes (Figure 3e) and fibroblasts (Figure 3f) displayed points of attachment to the fibers, and some fibroblasts spanned adjacent parallel strands in the same layer. Between and around fibroblasts, a pericellular matrix of variable density was deposited, extending up to ~0.5 µm from the cell surface, with small-diameter collagen fibrils on the outer edge of this matrix (Figure 3g,h).

## 4. Discussion

The approach of culturing a layered tissue using stacked layers of a pre-seeded electrospun mesh had several advantages. The most important advantage was that of reducing the time taken to construct a tissue in vitro for potential use as a graft. In this study, each layer was created simultaneously over 48 h and then the layers with attached cells on both sides of the electrospun mesh were stacked to instantly create a layered tissue which was then cultured for a further period of 5 days. The layered skin, with keratinocytes on the upper layer, was mechanically coherent by a total of 7 days’ culture, important for manipulations in experimental and clinical settings. Notwithstanding limitations on sourcing and expanding autologous cells in a clinical setting [4], this represents a significant advantage over approaches that can take 2–4 weeks [5,6,14].

A second advantage was that the sparse and aligned fibers of the spun scaffolds promoted the rapid attainment, within 24 h, of the typical elongated phenotype of dermal fibroblasts. Fibroblasts attached and extended along the fibers, and where the spun fibers were parallel to each other, sheets of cells became similarly elongated and aligned. This arrangement of cells also mimicked that seen in normal dermis, where fibroblasts and associated collagen fibers are aligned in bundles that collectively form a complex three-dimensional weave. The method of stacking scaffolds also allowed for creation of a woven tissue, albeit without vertical elements, by selectively placing the individual scaffold layers in the stack according to the alignment of their spun fibers relative to adjacent layers, thus achieving alternating fiber angles at different depths. Stacking of all scaffolds with all fibers aligned in the same direction, of course, has the potential to create tissues such as tendons and ligaments.

A further advantage of culturing the dermal fibroblasts on scaffolds with a relatively sparse fiber density was that the individual cells were able to exert contractile forces along their long axis while attached to single fibers, shortening the fibers. It was also observed that over several days, most of the spun fibers with attached cells lost their straight parallel alignments and formed wave patterns, again indicative of the cell collectively exerting sufficient forces to relieve tension on the fibers. Tensional and compressive forces on and within tissues are known to be important determinants of cell synthetic profiles, especially for extracellular components [15]. For example, the segments of flexor tendons that change direction around bony prominences are subjected to compression and show a marked change not only in the shape of the tenocytes from elongated to round but a significant increase in synthesis and secretion of matrix proteoglycans, including a relative shift from dermatan to chondroitin sulphate glycosaminoglycan chains [16,17]. Experimentation with stackable scaffolds of different materials, fiber densities, and alignments could be used to determine the best combinations for different tissue matrices.

The ability to predetermine the alignment of cells and spun fibers in skin cultured using stacked layers may provide another advantage. The arrangement of collagen bundles and elastic fibers in the dermis is anisotropic, that is, the weave is more closed and less extensible along the lines of skin tension or Langer’s lines than at right angles to Langer’s lines along which the weave is more open and much more extensible. Incisions made parallel to Langer’s lines generally heal with minimum scarring as the wound margins and repair tissue are not subjected to disruptive tensile forces (10). The use of stacked layers to engineer a dermis that mimics the natural weave patterns may ameliorate hypertrophic scar formation where there is an opportunity to electively place the grafted skin to match lines of tension in adjacent normal skin.

The biodegradable fibers used in this study had a low content of lactide and a high content of glycolide, the latter more susceptible to breakdown. We observed that the fibers in the stacked layers began to increasingly break from 10 to 13 days in culture, consistent with the faster degradation of glycolide compared to lactide polymer. In vivo implanted co-polymer meshes with a molar ratio of 50:50 lactide/glycolide degrade faster than meshes with a molar ratio of 85:15 [11]. In this study, the molar ratio of 10:90 was chosen for the purpose of early degradation of fibers and the transfer of mechanical load from the spun fibers to the sheets of fibroblasts to stimulate extracellular matrix production. If required, the scaffold could be designed to persist for longer periods by increasing the content of lactide.

Despite the fine mesh on each of the layer frames, and the increasing fragility of fibers with time in culture, the layers were surprisingly easy to handle in culture and to assemble into the stacks. The stainless steel frames were easily handled by forceps for transfer to and from culture dishes and during the stacking procedure. Addition and changing of culture media during the culture of individual frames, however, did require comparatively gentle transfers of media without generation of jet streams that could damage the fine spun fibers.

By design, the electrospun fibers occupied a small volume fraction of the total tissue mass (Figure 3d). A low content was considered important for minimizing the inflammation that accompanies the degradation of the PLG fibers, although reports indicate that inflammation associated with breakdown of fibers is not a major problem [11,18,19]. Nevertheless, even a low level of inflammation is associated with accumulation of a hydrated matrix containing hyaluronan and versican which can affect the fibrous composition of the extracellular matrix [7,9], notably small-diameter collagen fibrils and inhibition of assembly of elastic fibers [20,21]. A pericellular matrix of hyaluronan and versican is also a characteristic feature of fibroblasts in culture [22] and reducing production of these proinflammatory molecules, for example, reducing chondroitin sulphate, is known to stimulate formation of a fibrous matrix [23]. Interestingly, anti-inflammatory agents can be incorporated into degradable lactide/glycolide suture fibers [24], a strategy that may assist in early production of an extracellular matrix with mechanical integrity.

Our method of stacking layers of electrospun fibers, allowing for rapid construction of layered tissues, need not be restricted to the fiber composition used here. Cells attach to a variety of materials, including non-absorbable PBA (data not presented), and specific protocols could be developed depending on the intended final product. The method has the potential to be adapted to fiber materials of choice, to best suit the tissue engineered product. While the utility of this new method for culturing skin grafts for in vivo use remains to be determined, it does offer a strategy for testing other electrospun materials [25] and other cell types including stem cells [26] for the creation of layered tissues. Three-dimensional printing of multiple scaffolds that include interspersed cells [4,27] may also be adaptable to layered tissue construction. The layer frame approach may be adaptable to the engineering of complex tissues such as the myocardium where the challenge is to mimic the regional variations in fiber directions [28].

In summary, this study has demonstrated that the strategy of seeding multiple thin layers of aligned electrospun lactide-co-glycolide fibers with skin cells, then stacking the layers to form a coherent multilayered sheet, allows for construction of cultured skin over a period of one week. Further, the method of stacking allows for alternate placement of layers to achieve variable fiber orientations in the different layers, which, in turn, guide the orientations of cells and create a tissue that more closely resembles normal skin. The method has the potential to be adapted to a variety of layered tissues.

## Data Availability

The data presented in this study are available on request from the corresponding author.

## Dedication

We dedicate this paper to our colleague, friend, and co-author Neil (Cornelis Jacobus) Buunk who died unexpectedly in the final stages of the investigation. Neil’s electrospinning expertise and inventive skills in developing the means to controllably deposit aligned electrospun fibers onto the support frames were vital to the project and its success.

## References

[1] Ferreira M.C., Paggiaro A.O., Isaac C., Teixeira Neto N., Santos G.B.D. (2011). Skin substitutes: Current concepts and a new classification system. Rev. Brasil. Cir. Plást..

[2] Vyas K.S., Vasconez H.C. (2014). Wound Healing: Biologics, Skin Substitutes, Biomembranes and Scaffolds. Healthcare.

[3] Debels H., Hamdi M., Abberton K., Morrison W. (2015). Dermal matrices and bioengineered skin substitutes: A critical review of current options. Plast. Reconstruct. Surg. Glob. Open.

[4] Yu J.R., Navarro J., Coburn J.C., Mahadik B., Molnar J., Holmes IV J.H., Nam A.J., Fisher J.P. (2019). Current and future perspectives on skin tissue engineering: Key features of biomedical research. Translational assessment, and clinical application. Adv. Healthcare Mater..

[5] Boyce S.T., Warden G.D. (2002). Principles and practices for treatment of cutaneous wounds with cultured skin substitutes. Am. J. Surg..

[6] Böttcher-Haberzeth S., Biedermann T., Reichmann E. (2010). Tissue engineering of skin. Burns.

[7] Wight T.N., Kang I., Merrilees M.J. (2014). Versican and the control of inflammation. Matrix Biol..

[8] Birk D.E., Linsenmayer T.F. (1994). Collagen fibril assembly, deposition, and organization into tissue-specific matrices. Extracell. Matrix Assemb. Struct..

[9] Wight T.N., Kinsella M.G., Evanko S.P., Potter-Perigo S., Merrilees M.J. (2014). Versican and the regulation of cell phenotype in disease. Biochim. Biophys. Acta (BBA) General Subj..

[10] Flint M.H. (1976). The biological basis of Langer’s lines. The Ultrastructure of Collagen.

[11] Blackwood K.A., McKean R., Canton I., Freeman C.O., Franklin K.L., Daryl Cole D., Brook I., Farthing P., Rimmer S., Haycock J.W. (2008). Development of biodegradable electrospun scaffolds for dermal replacement. Biomaterials.

[12] Wang J.H.-C., Jia F., Gilbert T.W., Woo S.L.-Y. (2003). Cell orientation determines the alignment of cell-produced collagenous matrix. J. Biomech..

[13] Nurfaizey A.H., Stanger J., Tucker N., Buunk N., Wallace A., Staiger M.P. (2012). Manipulation of electrospun fibers in flight: The principle of superposition of electric fields as a control method. J. Mater. Sci..

[14] Pontiggia L., Klar A., Böttcher-Haberzeth S., Biedermann T., Meuli M., Reichmann E. (2013). Optimizing in vitro culture conditions leads to a significantly shorter production time of human dermo-epidermal skin substitutes. Pediatric Surg. Int..

[15] Gooch K.J., Tennant C.J., Gooch K.J., Tennant C.J. (1997). Mechanical Forces: Their Effects on Cells and Tissues.

[16] Merrilees M.J., Flint M.H. (1980). Ultrastructural study of tension and pressure zones in a rabbit flexor tendon. Am. J. Ana..

[17] Flint M.H., Gillard G.C., Merrilees M.J., Parry D.A.D., Creamer L.K. (1980). The effect of local physical environmental factors on connective tissue organization and glycosaminoglycan synthesis. Fibrous Proteins: Scientific, Industrial and Medical Aspects.

[18] Holzheimer R.G. (2005). Adverse events of sutures: Possible interactions of biomaterials. Eur. J. Med. Res..

[19] Athanasiou K.A., Niederauer G.G., Agrawal C.M. (1996). Sterilization, toxicity, biocompatibility and clinical applications of polylactic acid/polyglycolic acid copolymers. Biomaterials.

[20] Merrilees M.J., Lemire J.M., Fischer J.W., Kinsella M.G., Braun K.R., Clowes A.W., Wight T.N. (2002). Retrovirally mediated overexpression of versican v3 by arterial smooth muscle cells induces tropoelastin synthesis and elastic fiber formation in vitro and in neointima after vascular injury. Circulat. Res..

[21] Merrilees M.J., Beaumont B.W., Braun K.R., Thomas A.C., Kang I., Hinek A., Passi A., Wight T.N. (2011). Neointima formed by arterial smooth muscle cells expressing versican variant V3 is resistant to lipid and macrophage accumulation. Arterioscl. Throm. Vascul. Biol..

[22] Evanko S.P., Potter-Perigo S., Bollyky P.L., Nepom G.T., Wight T.N. (2012). Hyaluronan and versican in the control of human T-lymphocyte adhesion and migration. Matrix Biol..

[23] Hinek A., Boyle J.M., Rabinovitch M. (1992). Vascular smooth muscle cell detachment from elastin and migration through elastic laminae is promoted by chondroitin sulfate-induced “shedding” of the 67-kDa cell surface elastin binding protein. Exp. Cell Res..

[24] Lee D.H., Kwon T.Y., Kim K.H., Kwon S.T., Cho D.H., Jang S.H., Son J.S., Lee K.B. (2014). Anti-inflammatory drug releasing absorbable surgical sutures using poly (lactic-co-glycolic acid) particle carriers. Polymer. Bullet..

[25] Sundaramurthi D., Krishnan U.M., Sethuraman S. (2014). Electrospun nanofibers as scaffolds for skin tissue engineering. Polymer Rev..

[26] Gizaw M., Faglie A., Pieper M., Poudel S., Chou S.-F. (2019). The role of electrospun fiber scaffolds in stem cell therapy for skin tissue regeneration. Med. One.

[27] Singh D., Singh D., Han S.S. (2016). 3D printing of scaffold for cells delivery: Advances in skin tissue engineering. Polymers.

[28] Nguyen-Truong M., Li Y.V., Wang Z. (2020). Mechanical Considerations of Electrospun Scaffolds for Myocardial Tissue and Regenerative Engineering. Bioengineering.

